# Burns and Their Treatment*Read before the Buffalo Medical Club, December 13, 1882.

**Published:** 1883-01

**Authors:** Chas. A. Wall


### Burns and their Treatment.*

By Chas. A. Wall, M. D.

The treatment of burns and scalds is of interest to the general practitioner, both from their frequency and great fatality. Holmes ranks them, beyond all comparison, the most commonly fatal injuries which occur in civil life. In modern times, the large increase in the application of steam to all mechanical industries, and for the purpose of warming public buildings and dwellings, and the necessity thus engendered for intense heating furnaces, has vastly added to the number and severity of burns. Agnew also places kerosene oil among the most common causes, and recommends stringent regulations as to its use. In this country 6000 deaths from burns, yearly, is probably within the limit (there were 4266 deaths in 1860), while in England and Wales alone over 3000 occur in the same period. These facts compel all practicing physicians and surgeons to bestow some attention upon the subject. Burns are common to all periods and conditions of men and have been recognized from the most ancient times, so it is not strange to find that surgical writers of every age have given them considerable space in their treatises, while the mere compilation of the various authors, who have communicated their views to current periodic literature, would occupy more time than I have at my disposal. Notwithstanding this multiplication of authorities, there is, I think, little difference in the generally acknowledged effects, symptoms, prognosis or pathology, and it is only when we come to the treatment that any great diversity of opinion prevails.

Burns and scalds are in their nature the same, and only differ in the mode in which the heat is applied; a burn being caused by dry, a scald by moist heat. In using the term burns, therefore, I am to be understood as including both.

The effects of burns are local and constitutional, and vary according to the depth, extent and part involved, and to the character of the body producing the injury. Thus, an article

* Read before the Buffalo Medical Club, December 13, 1882.

having intense heat, may produce less disorganization of tissue by a momentary contact than one of vastly lower temperature, applied for some time; and a fluid will be apt to attack a greater extent than a solid.

The local effects have been divided by most writers according to the degree of tissue destroyed. Dupuytren made six classes, and these have been adopted by many surgeons (Holmes, Bryant, Ashurst, Velpeau, Erichsen). He includes, under the first, slight burns, causing redness, and followed by desquamation of the cuticle. In the second class, the injury is deeper, causing cutaneous inflammation, with the separation of the epidermis as vesicles of greater or less extent. In the third class, the skin is destroyed to and including the papillæ. In the fourth, the entire thickness of the skin is disorganized. The fifth class embraces those where the subcutaneous tissues and muscles are involved, while in the sixth class the whole thickness of the part is carbonized. This classification is, in some respects, convenient, but the differential diagnoses between severe burns of the third class, and those of the fourth where the skin is destroyed but no deeper parts injured, is almost impossible in many instances. Dupuytren calls attention to the eschar formed, that in the latter it is harder and darker in color, and especially to the fact that, upon pressure, there is less pain where the nerve extremities are killed; but when we consider that a large proportion of burns are in children, little dependence can be placed upon this sign.

These distinctions, as given by Dupuytren, are thought by some authors to be too complex for practice and tending to confuse rather than to assist. Heister, Callisen, Samuel Cooper and Chelius give four classes. Gross recognizes but two, simple and complex. Druitt, Billroth, Stephen Smith, Agnew and Morton all agree in making three classes. This division seems to be the most preferable, and is the one generally followed by the latest writers. The use and necessity of recognizing the relative depth of burns may be best seen in the treatment where, as Holmes says, the great diversity of remedies recommended is in great part due to the fact that surgeons do not remember that different degrees of burns require different treatment.

The three classes of burns, now usually accepted, are:

First, burns causing redness followed by desquamation of the cuticle. They are of little importance and do not fall under the care of the surgeon unless a considerable extent of surface is involved, in which case they may be serious and endanger life. Burns of equal depth differ from each other in a greater ratio than the extent of the surface affected, and it is well to bear this in mind when judging of the prognosis.

The second comprises those burns which form vesicles, whether they are followed by ulcers or not. The entire thickness of the skin is not destroyed, and if an ulcer forms, it is never deep and granulation soon restores the skin to its normal condition.

The third class includes burns in which an eschar is formed and thrown off by subsequent suppuration. They vary in depth, but all produce death in the tissue destroyed, whether it be the skin alone or extend to the carbonization of the part wounded. These deepest injuries of the third class, which correspond to the sixth class of Dupuytren, are seldom seen and can occur only on the extremities.

While the three classes are usually well marked and may, in some cases, be found on the same person, it is not uncommon for them all to occur in one wound, due to the difference of temperature in the body producing the burn.

The constitutional effects differ according to the depth and extent of the injury, and also to its position. A burn upon the chest, of little depth, may produce more constitutional effects than the entire destruction of a hand or foot. Three well marked stages are found in all serious burns. First, that of depression, which lasts from the reception of the injury until reaction takes place, and is dangerous in extensive burns, even if their depth be inconsiderable. Many die from shock alone, or from shock combined with pain. Of two hundred fatal cases, from various sources, over one-half died during the first forty-eight hours, some not having reacted at all and others only slightly. This stage lasts about two days, when reaction having been established, the second begins, which may be accompanied by severe fever and delirium. Here we must watch for internal congestion, inflammation of the brain, lungs, or alimentary canal. The local influence of the injury is marked, a burn of the abdomen being often followed by peritonitis or enteritis, while inflammation of the bronchi, lungs or pleuræ follows a wound on the chest. It is not so well marked in burns of the head. Curling, in 1842, called attention to the fact that ulceration of the duodenum is often met with in children. This is a grave complication, and proves fatal, either from hemorrhage or perforation and peritonitis. He thought it was due to the vicarious action of Brunner's glands. Feltz, however, considered it to be caused by capillary embolism. It probably arises from congestion, as do the other internal complications (Holmes, Agnew); but why other portions of the intestines are not equally liable is unknown.

In about two weeks the eschars separate, when the third, or stage of exhaustion sets in. The patient now suffers from extensive ulceration, and may sink under it and die from the profuse suppuration. Septicæmia and visceral degeneration may bring about a fatal result in other cases.

The local signs of burns are well known. They are the physical result of heat applied to organized tissue, and vary from the faintest browning to the black carbonization of tissue. The local symptoms are those of inflammation, the same as from any other cause, and are proportionate to the depth and extent of the injury. Ulcerization, granulation and cicatrization do not differ from that seen in the general repair of the continuity of the skin.

The constitutional symptoms are those of shock, pain, and coldness of the surface and extremities. This may be transient in small burns, but in the more serious it is well marked.

We will then find the patient shivering, the surface pale and cold, the pulse quick and feeble, and the depression, in some cases, amounting to total collapse, from which they never react and die in a few hours. After reaction comes on, there will follow fever, with symptoms of internal congestion and inflammation when complications occur. If the fever is high or continued, albumen will usually be found in the urine. Pain often continues unabated. During the third stage the symptoms are those of exhaustion and debility, while little pain is felt except when dressing the wounds.

The pathology of burns is that of congestion and visceral engorgement, as has been shown by Holmes and Erichsen. How much this may be owing to change in the blood corpuscles is under dispute. The later manifestations are those which naturally result from this condition.

If a burn be of great extent, involving a large proportion of the body, the danger to life is extreme. Practical observations have fixed as an axiom that when more than one-half the body is injured, death will result. A majority of the deaths take place during the first stage, without complete reaction having been brought about. Scalds in children are often rendered dangerous by the inhalation of steam causing acute inflammation of the air passages, with death from œdema and spasm of the glottis. It is commonly accepted among the laity that in conflagrations death follows from inhaling flame. It is hardly necessary to say that it is due to smoke and foul or noxious air. During the second stage death occurs from any of the internal complications mentioned, while later the danger arises from exhaustion, debility, septicæmia, and visceral degeneration.

The constitutional treatment of burns is, during the first stage, directed to the depression and pain. Reaction must be brought about, and this is best done by administering a full dose of opium, combined with brandy, to be repeated, if necessary, in an hour or two. According to Dr. John Morris, alcoholic drinks are not advisable. The best stimulant is strong, hot coffee, to which a little brandy may be added, if manifestly needed. The dose of the anodyne must be double the ordinary one, and propor.ionate to the age and condition of the patient (Gross). Chloroform, while dressing the burns, may be indicated in some cases. Warmth to the surface is soothing and assists in promoting reaction. If any part is absolutely destroyed, it can be removed at once, but nothing should be lost by too early interference. Bryant relates a case where a man stepped into molten lead, and his foot, when withdrawn, was encased in a boot of metal and the tissues destroyed to the bone. The foot was amputated above the ankle without disturbing the metal. Injuries of this nature can only be seen upon the extremities, and it is usually best, except in extreme cases, to be a little conservative. In injuries in which a large surface is denuded of cuticle, the exudation may be so great as to endanger life and require surgical interference and the removal of an arm or leg involved. In the second stage thirst must be quenched with ice, as the vomiting induced by flooding the stomach with fluid is an additional danger. Constipation is to be relieved by a simple saline or mild laxative. Opium must be continued where there is pain or irritability. After suppuration begins, stimulating and sustaining food must be given in quantity. In some cases the amount required is astonishingly large. Tonics may also be used with great benefit, especially combinations of bark and iron. Internal complications must be met as they arise.

The local treatment of burns is the most diversified of all surgical injuries. Nearly every author, and, indeed, almost every practitioner has his own peculiar method, which he recommends as beyond comparison the best to be employed. Holmes, in his excellent monograph, says: "The local treatment of burns is a subject on which many books have been written, and perhaps more numerous remedies recommended than in any other branch of surgery." It would be interesting to study the treatment of burns in the earlier periods,* but it is beyond the limits of this paper to give even a classified table of the remedies advised, and it will be necessary to confine ourselves to a general view of some of the most important methods in use at the present time.

Holmes excludes the air from the burn by some local application, such as flour, starch, carron oil, or cotton wool, where the injury is slight, but when the skin is destroyed, considers nothing better than oil of turpentine. He gives as a good stimulating salve for deeper burns,

℞ Unguenti elemi, ℔j,

Unguenti lambuei, ℥ij,

Copaibæ, ℥iij.

M.

and applies, when the discharge is great,

℞ Acidi carbol., ʒj,

Olei olivæ, ℥iij.

M.

removing all sloughs when they separate. Burns should not be dressed too frequently, but care should be taken to guard against accumulation of pus and fetor. When blisters rise, he opens them at once and allows the fluid to escape, but preserves the skin, as it forms a protection for the part. This is the almost universal practice, and authors lay the greatest stress upon it, and recommend that any clothing, immediately covering the part, be cut off in removing to escape the danger of rupturing the blister. These may, in some instances, be of great extent, a complete cast of the hand and arm having been thrown off in one case. James Syme, however, opposes retaining the epidermis, when separated. He says: "When blisters rise, the detached cuticle should be not only laid freely open, but taken away altogether, as its pressure seems to increase the irritation."

* Hippocrates recommended, lard and oils or their combination with astringents. So late a writer as Dr. Packard places the utmost confidence in the use of *fresh lard*, no great departure from the earliest treatment.

I have seen one case in which this procedure seemed to give a good result. Willie O. was burnt on the face and extremities, and, by violently clasping his hand to his face, ruptured the blisters. I carefully removed the shreds from his forehead, nose, cheeks and chin, and applied a solution of bicarbonate of soda, but was obliged, later, to change to carron oil, from the impossibility of retaining the former in place. The injury to his face healed quickly, and the ulcers, although deep in places, closed without leaving any cicatrix. On the extremities the burns were of a less degree and the cuticle was not removed, but punctured, still the cure was delayed for some time after that upon the face.

Syme declares that carron oil is now entirely superceded, and says: "If the part retains its vitality, cold and cotton will prove most useful, and if the texture of the part be destroyed, nothing is better than a poultice until the sloughs separate, and then the treatment is the same for both." And again: "Cold applications afford most relief, and sometimes, if at once employed, prevent inflammation." John Earle treated burns with ice and saw no bad results following. He, however, limited its use to cases having no contra-indication, such as depression or internal congestion.

The plan introduced by E. Kentish early in the present century is, first to wash the part in oil of turpentine and then dress it with resin ointment, thinned with turpentine.

℞ Ung. resinæ, ℥j,

Olei terebinth. ℥ss.

M.

Applied on lint.

Druitt favors carron oil, water dressings, or poppy solution, and when ulcers occur, uses chalk ointment or zinc lotion. In large injuries, where there is no tendency to heal, he covers the ulcer with bismuth and makes pressure with sheet lead, held down by adhesive plaster.

Thomas Bryant applies to burns of small extent carbolic oil, as :

℞ Carbolic acid, ℥j,

Olive or linseed oil, Oj.

M.

or ointment,

℞ Carbolic acid, ʒiv,

Lard, ℥iv,

Castor oil, ℥j.

M.

When larger surfaces are involved, these should be replaced by carron oil or co. tinct. iodine ʒj to water Oj. He thinks that when the ulcer is large, too much patience can not be exercised in attempting skin grafting, and any surgeon who does not thoroughly try it, is negligent of his duty.

Cold applications, according to Prof. Gross, should not be resorted to without great care, from fear of internal congestion and effusion and that they are chiefly adapted to the young and strong during summer, and even then there is danger. He thinks any of the domestic remedies are good, but has long exclusively used carbonate of lead in the form of white lead of the consistency of cream. He has never seen any manifestations of lead poisoning following its exhibition. He considers carron oil so exceedingly filthy and disgusting that it should be discarded from private practice.

Dr. Morris condemns carron oil as useless. He recommends

℞ Acidi carbolici, fʒj–iv,
Morph. sulph., gr. ij,
Olei olivæ, f℥vj.
M.

a suitable application for most burns. In bad scalds of children as he places them in a bed of bran. This has the advantage of absorbing all exudation and need not be changed for some time.

Erichsen protects the part with any of the usual remedies and has found carron oil to agree best in some cases. He considers it of the greatest importance that the dressing be changed as seldom as possible.

Samuel Cooper called lime water and oil inert, and strongly recommended the Kentish plan. To repress the growth of exuberant granulations and absorb secretion he employed powdered chalk.

R. Liston applied cold to trifling burns, and to more serious ones, watery acids or alkalies, either hot or cold, and found good results from each, as he also did from flour, tar and pitch.

Prof. Billroth recommends, when all three degrees of burns are combined, nitrate of silver gr. x to aquæ ℥j to be painted over the parts, and compresses wet with it to be constantly applied until the eschar separates. The pain from this cauterization is sometimes very severe, but a crust soon forms and the pain then ceases entirely. In burns of the first and second class the treatment looks more towards alleviation of pain than to any other end. Remedies are used which cover the skin and protect it from the air. He does not like collodion from its tendency to crack, and at these points the skin will become sore. Velpeau found that pressure by adhesive straps will promote cicatrization.

Dr. Marsh uses a saturated solution of alum, ℥j to water ℥viij, applied on cloths to recent burns.

Dr. Ely McClelland, U. S. A., says that a solution of bicarbonate of soda instantly relieves pain and prevents cicatricial deformity. He uses a saturated solution, one-half pound to a quart of water, applied on patent lint. The dressing should not be disturbed for several days, and must never be allowed to become dry.

Dr. A. H. Buckmeister gives as an improvement on the last: "To equal parts of linseed oil and water to which lime has been added (making it about three times the strength of aquæ calcis) there is placed enough sodium bicarbonate to make a thick paste (in severe cases morphia may be added); this mass is applied with loose roller bandages in the usual way. This dressing has all the advantages of the sodium bicarbonate alone and does not adhere to the skin."

The following has been going the rounds of the journals for the last few months: "Saturate a soft piece of fabric with alcohol, lay it over the burn, then cover it with cotton or finely picked oakum. This is the most cleanly dressing that can be adopted. It may be thought that alcohol, applied to a burn, will produce more pain; but try it and you will be agreeably surprised to observe how quickly it will allay the pain. Subsequently, disturb the dressing as little as possible; wet the dressing with alcohol, and the result you will find better than by any other method."

J. B. C. Guzzo has found glycerine most valuable in preventing scars. He uses it diluted with an equal quantity of water, or pure, according to the nature of the burn, and applies it on linen compresses. He also uses a combination of one part of glycerine with three parts of collodion, applied with a small hair brush. The results of the employment of glycerine in this manner have convinced him that it has some specific action in expediting the cicatrization of burns, however extensive they may be.

Ashurst does not believe that, with regard to local applications, it makes a great deal of difference what article is used, provided the surface is thoroughly excluded from the air. More important than the particular article used is the mode of using it. Only a small portion of the surface should be uncovered at once, and the burn, if extensive, should thus be dressed, as it were, in detachments. When the sloughs have separated, the remaining ulcer may be dressed with lime water, dilute alcohol, or zinc or resin ointment, as in the case of any other granulating surface. Care must be taken to guard against undue contraction of cicatrix by the use of appropriate splints and bandages.

W. R. E. Smith cleanses the parts after suppuration has begun, then washes with carbolated oil and dusts from a flourdredger,

℞ Zinci oxidi,
Magnesiæ carbonatis, aa, ℥j,
Pulveris amyli, ℥ij.
M.

This forms a firm incrustation like a scab, under the protection of which the parts heal rapidly. It should be applied whenever the moisture appears.

Dr. Binkerd gives, as a soothing anæsthetic ointment,

℞ Pulvis iodoformis, ℈ij–iv,

Cerati, ℥j.

M.

Five or six drops of carbolic acid may be added.

Prof. J. Lister recommends, as a dressing for burns,

℞ Carbolic acid, ʒiv,

Olive oil, ℥iv,

Castor oil, ℥j.

M.

In extensive injuries, Hebra has found great benefit in the continued use of the water bath. It may, under proper circumstances, be continued for a week or more. Dr. Duga states that Labarraque's solution will immediately relieve pain and prevent suppuration when the whole thickness of the skin has not been destroyed.

Stephen Smith employs, in burns of the first class, cold water, lead water, scraped potatoes, flour, etc. In those of the second class he uses carron oil, carbolized oil, white lead or zinc ointment. While in burns of the third class he first dresses them to relieve pain and then afterwards so dresses them as to promote the separation of sloughs.

Dr. Agnew would treat burns of the first class with cold water, unless their extent was so great as to render its employment dangerous. In the larger burns he protects the skin with any of the common remedies. To those of the second class he applies benzoated zinc ointment and envelops the whole in cotton-batting. In burns of the third class, after a few days, he uses a lotion of lead water and laudanum to separate the sloughs, or for this may be substituted flaxseed poultices, changed every six or eight hours. As soon as suppuration is well established, the parts should be thoroughly cleaned with deodorizing washes, and the poultices rendered stimulating by sprinkling over them a quantity of the compound resin or Kentish ointment. After granulation begins, special attention must be given to the ulcer to prevent contraction. No medication will entirely prevent it, he thinks, but a solution of caustic potash, gr. iij to water ℥j, applied lightly over the granulations every day or two during the process of healing, lessens this tendency, to some degree.

The above citations might be multiplied without limit, but enough have been presented to show the general principles which underlie the various methods of treatment. From them we conclude: that all burns require to be protected from the air; that inflammation must be combatted, and any foreign body removed before granulation can begin. Applying these facts, we find that the first class being superficial and the inflammation unimportant, any substance that protects the skin will be sufficient. As dry materials cause less inconvenience than moist, they are usually preferred. In the second class the injury is more severe. Congestion and pain are prominent. Capillaries are engorged and nerve extremities exposed. The indications are to relieve the pain and lessen the inflammation. This is best done by local anodynes and moisture. We find in lotions, oils and ointments the best applications to both protect the parts and relax the irritated tissues, and add to them any remedy which will exert a local anodyne effect upon the sensitive nerves.

In burns of the third class the disorganized tissue acts as a foreign body and must be removed. This is performed by the use of poultices which soften the eschar and stimulate the surface of the wound to a healthy action. Any substance which will macerate the destroyed part and incite the depressed function, is applicable. Oils have been most often used, but a poultice will answer by adding to it the stimulating property, if that be wanting.

The value of any dressing for general use in all burns will be in proportion to the degree in which it protects the skin, relieves pain and relaxes the inflamed tissues; and while they may all do this to a greater or less extent, it is better for us to meet the indications as they may present, and not be too much attached to a particular remedy.

